# Successful rescue of disseminated *Nocardia* infection with multiple abscesses in a patient with membranous nephropathy after cardiopulmonary resuscitation: A three-year follow-up

**DOI:** 10.7555/JBR.37.20230107

**Published:** 2024-01-25

**Authors:** Yili Xu, Hanyang Qian, Wen Qian, Li Dong, Weiying Liu, Yan Zhu, Yaning Mei, Yi Xu, Ling Wang, Yi Xia, Xu Qi, Huanping Mei, Xueqiang Xu, Huijuan Mao, Changying Xing, Ningning Wang

**Affiliations:** 1 Department of Nephrology, the First Affiliated Hospital of Nanjing Medical University, Jiangsu Province Hospital, Nanjing, Jiangsu 210029, China; 2 Department of Nephrology, Nanjing Tongren Hospital, Nanjing, Jiangsu 211102, China; 3 Department of Image, the First Affiliated Hospital of Nanjing Medical University, Jiangsu Province Hospital, Nanjing, Jiangsu 210029, China; 4 Department of Infectious Disease, the First Affiliated Hospital of Nanjing Medical University, Jiangsu Province Hospital, Nanjing, Jiangsu 210029, China; 5 Department of Nephrology, the Second Hospital of Nanjing, Nanjing, Jiangsu 210029, China; 6 Department of Microorganism, the First Affiliated Hospital of Nanjing Medical University, Jiangsu Province Hospital, Nanjing, Jiangsu 210029, China; 7 Department of Hematology, the First Affiliated Hospital of Nanjing Medical University, Jiangsu Province Hospital, Nanjing, Jiangsu 210029, China; 8 Department of Rheumatology and Immunology, the First Affiliated Hospital of Nanjing Medical University, Jiangsu Province Hospital, Nanjing, Jiangsu 210029, China

**Keywords:** *Nocardia* infection, glomerulonephritis, membranous nephrology, cardiopulmonary resuscitation

## Abstract

Nocardiosis manifests as an opportunistic infection, primarily affecting individuals who are immunocompromised and susceptible to the infection. We present a case study of one patient with nephrotic syndrome and membranous nephropathy, who underwent treatment with prednisone and cyclosporine in 2016. In early 2017, the patient was diagnosed with a "fungal infection" and discontinued the use of cyclosporine. After one month of anti-infection therapy, a cranial magnetic resonance imaging scan showed multiple abscesses in the right temporal region. The diagnosis of nocardiosis was confirmed based on the presence of metastatic abscess masses, multiple lung and brain lesions, and a positive culture of *Nocardia* in the drainage. We changed the anti-infection therapy to a combination of trimethoprim-sulfamethoxazole (TMP-SMX), minocycline, and voriconazole. However, the patient experienced a sudden cardiac arrest and subsequently recovered after cardiopulmonary resuscitation. During the five-month follow-up period following the discharge, the patient displayed an enhanced nutritional status and stable renal function. The focal infection ultimately resolved during the subsequent three years. Neuro-infection caused by *Nocardia* should be considered in immunocompromised patients, and TMP-SMX is the preferred initial therapy; however, because of the high mortality rate, a long-term combination therapy with imipenem, cefotaxime, amikacin, and TMP-SMX is suggested.

## Introduction

*Nocardia* is a genus of aerobic Gram-positive actinomycetes, which is currently composed of approximately 100 species. The infection caused by *Nocardia*, known as nocardiosis, is commonly regarded as an opportunistic infection^[1]^. Nocardiosis ranges from the skin and soft tissue infections to systemic dissemination, such as to the respiratory tract and central nervous system (CNS), particularly in immunocompromised patients. The mortality rate ranges from 20% to 30%. In cases where the CNS is involved, this rate can escalate to 50%^[2]^.

Here, we report a patient diagnosed with membranous nephropathy accompanied by nocardiosis affecting the CNS, respiratory system, and soft tissues. We highlight the successful management of this infection, along with improvement in their nutritional status and absence of any renal dysfunction. The protocol was established according to ethical guidelines of the Helsinki Declaration and was approved by the Institutional Review Board of the First Affiliated Hospital of Nanjing Medical University (Jiangsu Province Hospital). A written informed consent was obtained from the patient for publication of this case report and any accompanying images.

## Case presentation

The patient was a 43-year-old female with a history of diabetes and hypertension for one year. She first visited our hospital and received a diagnosis of nephrotic syndrome (NS) in April 2016. A renal biopsy revealed membranous nephropathy at stage Ⅱ–Ⅲ. An immunomodulatory therapy was initiated on May 5, 2016, with a daily dose of 30 mg of prednisone and a twice-daily dose of 50 mg of cyclosporine. After experiencing transaminase elevation in August 2016, the patient's prednisone medication was switched to an equivalent dose of methylprednisolone. The patient presented to the hospital between January and March, 2017, with symptoms of cough and fever, and was diagnosed with "suspected pulmonary fungal infection". The cyclosporine medication to the patient was ceased, and multiple antibiotics were administered for three weeks, but without a significant improvement. After an anti-fungal therapy, lung infection of the patient improved, and the fever was controlled. The detailed therapeutic schedule is shown in ***Supplementary Table 1*** (available online). However, on March 20, 2017, the patient observed a lump on her right buttock that rapidly grew in size from that of a bean to that of an egg, with pain but no redness or swelling (***Supplementary Fig. 1A***, available online). Subsequently, a similar lump appeared in popliteal fossa of the right leg (***Supplementary Fig. 1B***, available online). On March 24, 2017, the patient was admitted to our hospital for treatment that included the administration of "methylprednisolone 16 mg qd" and "voriconazole 100 mg bid". The patient occasionally coughed up white mucous sputum but did not experience a fever. From April 2016 to March 2017, the patient lost approximately 25 kg in weight (her weight was 36 kg, height was 150 cm, and body mass index [BMI] was 16 kg/m^2^ in March 2017).

**Figure 1:**
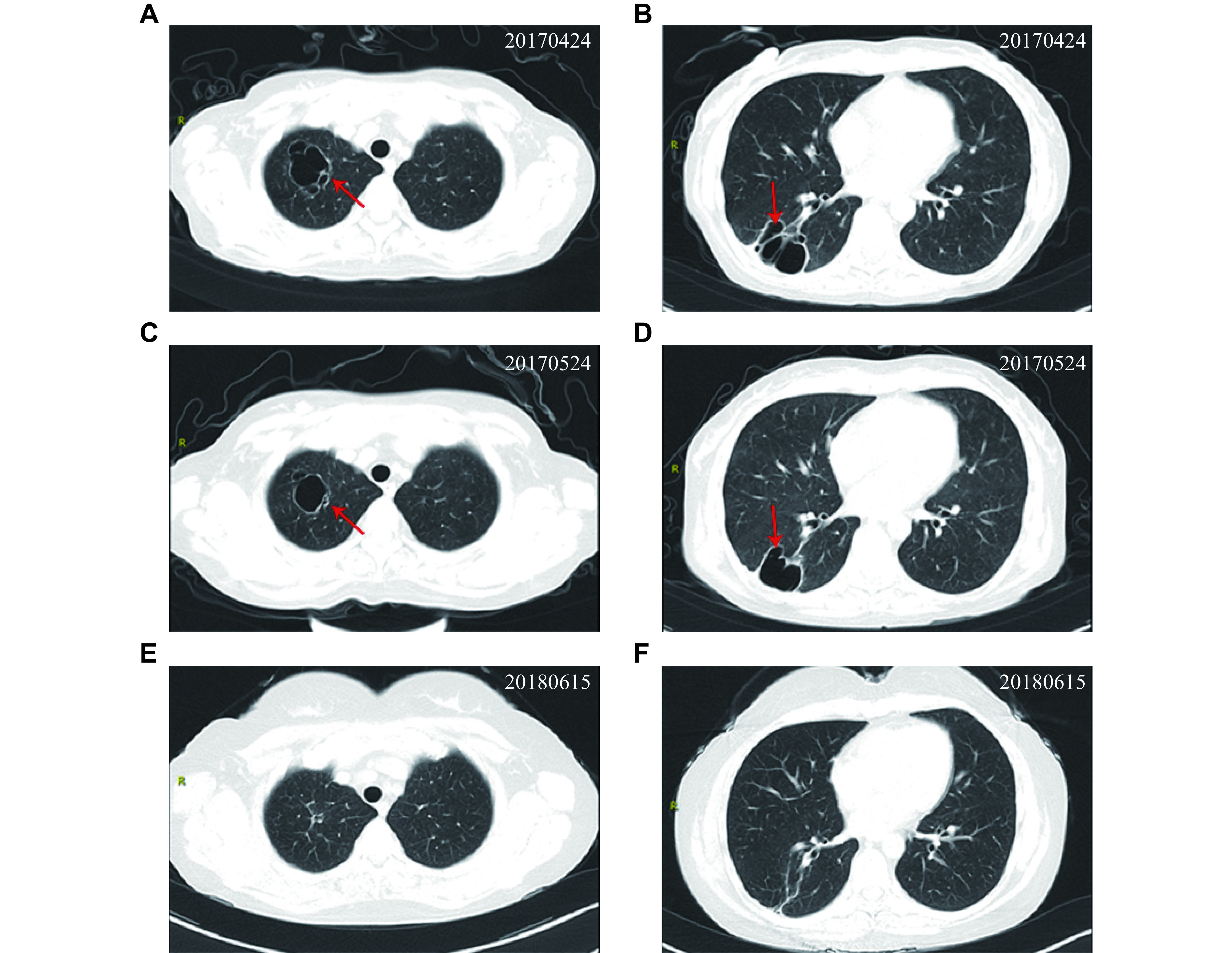
Pulmonary bullae with infection were absorbed during the follow-up.

**Figure 2:**
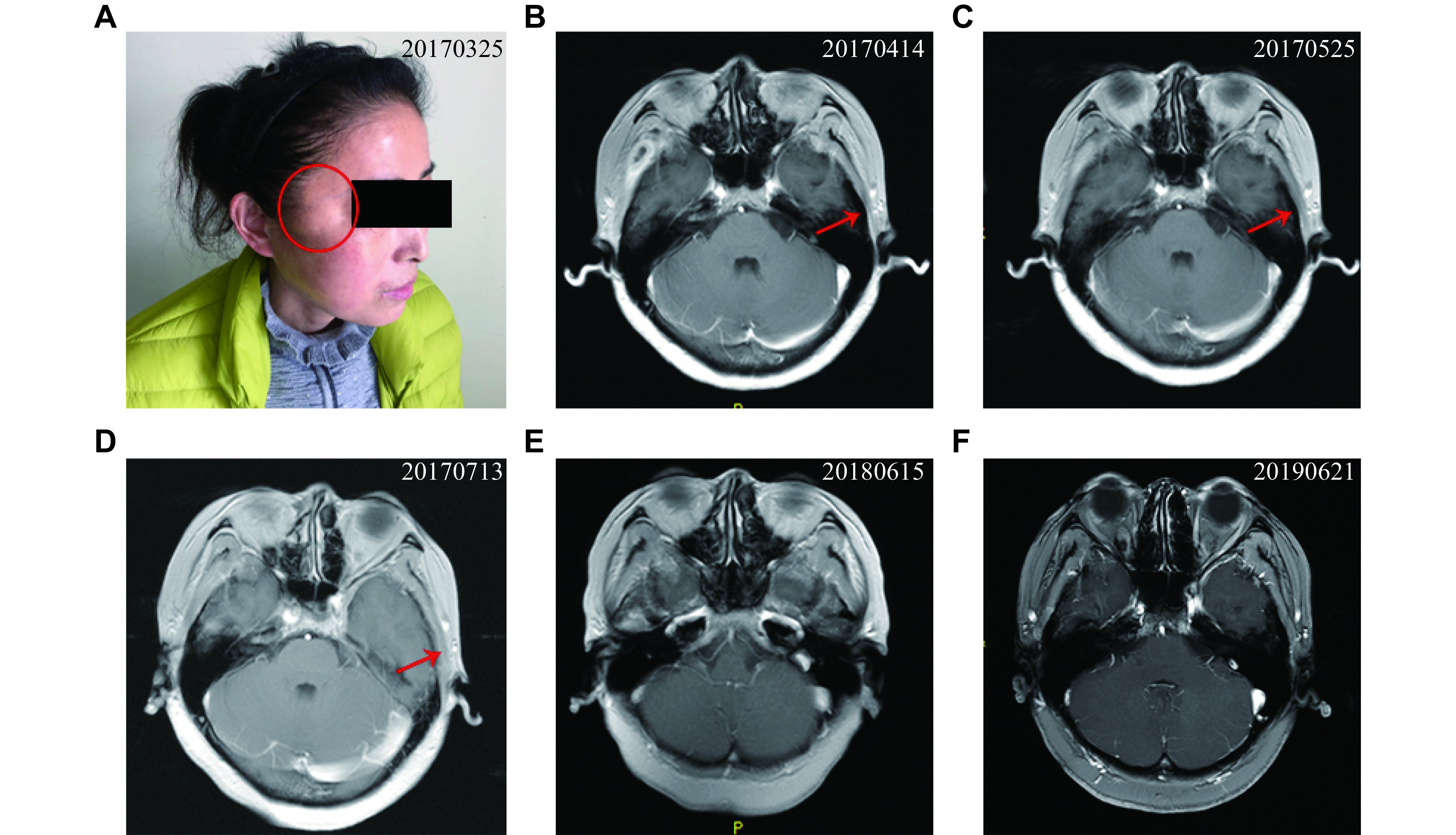
Magnetic resonance imaging (MRI) views revealed an oval lesion located within the right temporal muscle.

**Figure 3:**
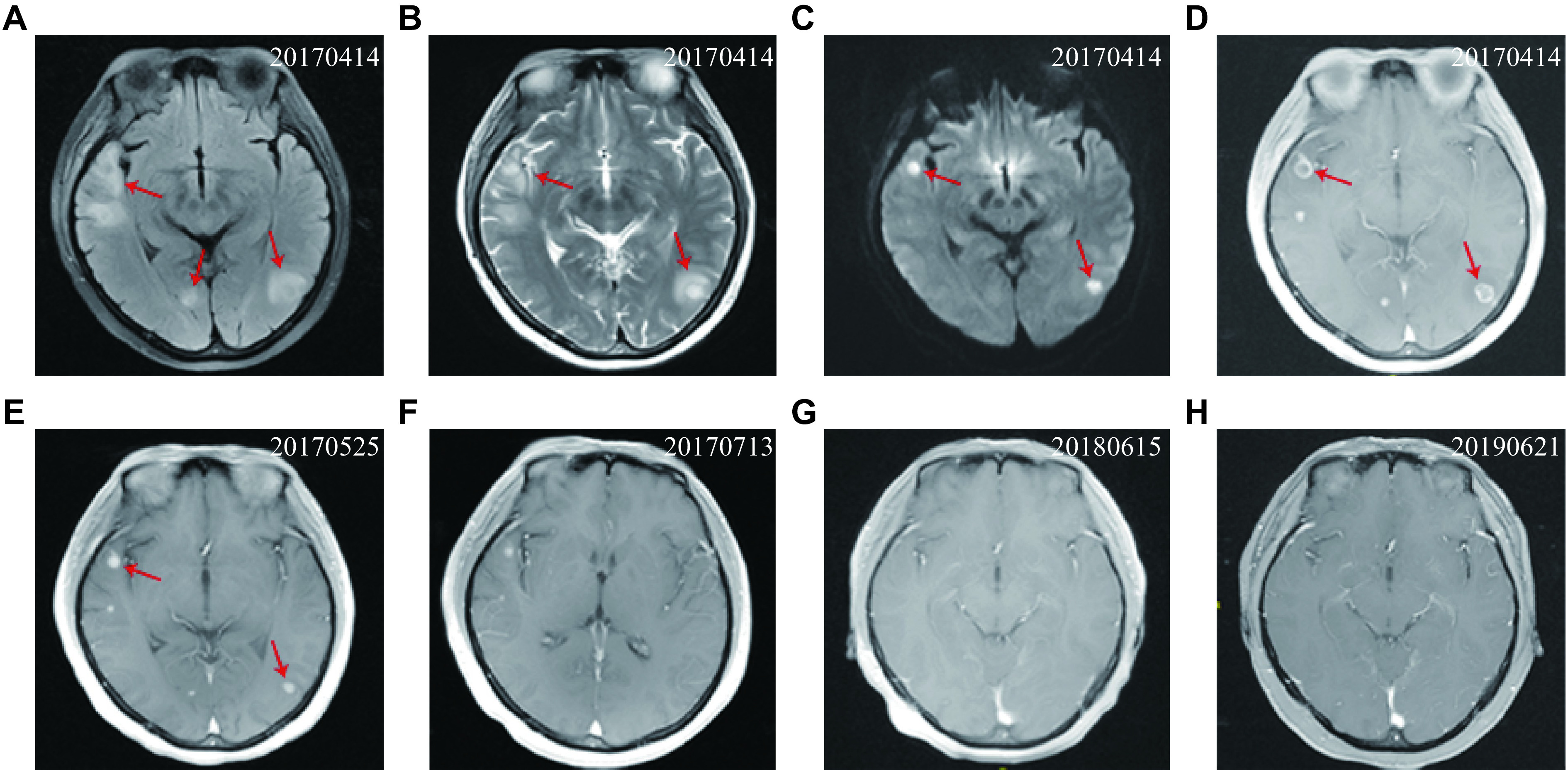
Magnetic resonance imaging (MRI) views of the brain abscess before and during the follow-up.

Laboratory examination revealed as follows: white blood cell count, 10.45 × 10^9^/L; serum C-reaction protein (CRP), 21.36 mg/dL; serum creatinine (SCr), 32.1 µmol/L; urinary microalbumin/urinary creatinine, 769.1 mg/g; 24-h urinary protein, 858.40 mg; endogenous creatinine clearance rate, 111.7 mL/min. Chest computed tomography (CT) revealed a cystic translucent shadow fused with a slightly thickened capsule wall (***Fig. 1A*** and ***1B***), and this fusion was observed in the right upper and lower lobes of the lung, surrounded by a patchy cord-like shadow. The day following hospitalization, a growing mass was discovered in the right temporal region and exhibited a rapid increase in size (***Fig. 2A***). To identify the mass observed in the right temporal region, cranial magnetic resonance imaging (MRI) was conducted. The MRI displayed an oval lesion with a ring enhancement in the right temporal muscle (***Fig. 2B***). Multiple concentric rims were observed on T2-weighted MRI, with the largest one situated in the right temporal lobe, measuring approximately one centimeter in diameter (***Fig. 3A***–***3D***).

A puncture was performed for etiological analysis on the third day after admission. The opaque pink gray effusions were drained percutaneously after intubation (***Supplementary Fig. 1C***, available online). Initial Gram staining revealed no microorganisms, and the blood cultures were negative for general anaerobic and aerobic bacteria. Based on the symptoms and medical history, a *Nocardia* culture was performed, which showed a positive result. The recommended treatment for pulmonary nocardiosis involves a sulfonamide-containing regimen. Therefore, starting from March 31, 2017, the antimicrobial therapy was adjusted to trimethoprim-sulfamethoxazole (TMP-SMX) administered orally twice a day, with the dosage consisted of 0.96 g in the morning and 0.48 g in the evening.

A multidisciplinary consultation, which included experts in nephrology, infection, respiratory, rheumatology and immunology, imaging, and neurosurgery, was organized. The dosage of methylprednisolone was reduced to 4 mg/day. Wide-spectrum antibiotics were replaced with oral TMP-SMX at a dosage of 0.96 g four times daily as well as intravenous minocycline at a dosage of 50 mg twice daily, linezolid at a dosage of 600 mg twice daily, and imipenem cilastatin sodium at a dosage of 1 g every eight hours. Glycerin fructose dehydration was utilized to mitigate the intracranial pressure (***Supplementary Table 1***, available online).

On the 20th day following admission (10:08 a.m., April 15, 2017), the patient experienced a coma, as well as a sudden respiratory and cardiac arrest. The patient's heart rate was restored after three minutes of cardiopulmonary resuscitation. At 10:22 a.m., the patient presented with epilepsy caused by brain edema. Following the treatment with 5 mg of diazepam to alleviate spasms and furosemide to lower intracranial pressure, the patient's vital signs stabilized within five minutes.

In addition to receiving anti-infective treatment, the patient recieved a symptomatic and supportive therapy, which included intravenous immunoglobulin, albumin, and nutritional support. On April 23, 2017, the dosage of TMP-SMX was reduced to 0.96 g three times a day because of a notable gastrointestinal reaction. On April 26, 2017, the patient's serum 1-3-β-D-glucan increased to 620.7 pg/mL, the CRP increased to 27.00 mg/L, and the procalcitonin level was 0.16 ng/mL; thus, voriconazole was orally administered as an antifungal treatment, and imipenem cilastatin sodium was discontinued.

The chest CT scan conducted on May 24, 2017 (***Fig. 1C*** and ***1D***) revealed the presence of infection-related bullae in upper lobe of the right lung. Additionally, more severe ones were observed in the lower lobe, which were absorbed slightly, compared with those in April 24, 2017. One month later, a cranial MRI revealed a significant decrease in the size of the brain abscess (***Fig. 3E***) and oval lesion located in the right temporal muscle (***Fig. 2C***), compared with those in April 24, 2017 (***Fig. 2B***).

After two months of the treatment, the patient's oval lesion further decreased (***Fig. 2D***), white blood cell counts, serum procalcitonin and CRP also decreased significantly, and the patient's general condition improved with infection control. The patient was discharged on June 9, 2017 with oral anti-infective drugs, including TMP-SMX 0.96 g tid, minocycline 100 mg bid, and voriconazole 100 mg bid.The patient was followed up from 2017 to 2019. The bullae, which were observed in lower lobe of the right lung on the chest CT scan conducted on June 15, 2018 (***Fig. 1E*** and ***1F***), showed a significant reduction, compared with the ones observed on May 24, 2017. Multiple ground glass density shadows were observed in the upper lobes of both lungs and the lower left lobe.

Neuroimaging (July 13, 2017, ***Fig. 3F***) revealed the number and size of brain lesions were further decreased, compared with those observed in MRI on March 25, 2017 (***Fig. 3E***). Six months later (October 10, 2017), the patient's SCr was 68.3 µmol/L and serum urea was 6.1 mmol/L, the 24-h urinary protein decreased to 58.5 mg, CRP decreased to 3.4 mg/dL, and weight increased to 50 kg, reflecting a weight gain of 15 kg with controlled fasting blood glucose levels (***Supplementary Fig. 2***, available online). The MRI conducted at the one-year mark (June 15, 2018, ***Fig. 2E*** and ***Fig. 3G***) and two years later (June 21, 2019, ***Fig. 2F*** and ***Fig. 3H***) revealed the absence of inflammatory tissues and purulent abscesses in the brain and within the right temporal muscle.

## Discussion

As early as 1962, Kerbel *et al*^[3]^ reported a case of a patient with nephrotic syndrome who was treated with high-dose glucocorticoids and contracted with *Nocardia* infection involving multiple organs, including the lungs, heart, liver, and adrenal gland. Here, we present a case study of a patient diagnosed with NS and diabetes, who was undergoing a long-term therapy with steroids and an immunosuppressant. Additionally, the patient was experiencing malnutrition. Therefore, it is imperative to strike a balance between the efficacy of steroid and immunosuppressant therapies, while also taking into account the risk of potential infections. Nocardiosis primarily occurs through inhalation, with the lungs being the organ the most commonly affected. Clinical manifestations of pulmonary involvement include pneumonia, lung abscess, pleural effusion, empyema, and cavitary disease. In patients presenting with concurrent pulmonary symptoms, it is advisable to consider conducting an evaluation for *Nocardia* infection. The potential for rare nocardiosis should be taken into consideration in patients experiencing fever, cough, or expectoration, who do not respond to a standard antibiotic treatment, and whose chest imaging reveals nodules or masses located close to the pleura.

Nocardiosis can disseminate to the CNS in approximately 30% of the cases and result in brain abscess in 20% of the cases. In 2016, the General Hospital of Nanjing Military Region reported that nine patients were infected by *Nocardia* presented with NS, and that all individuals were presented with pulmonary nocardiosis, with one case specifically involving the brain, a rare occurrence exhibiting an elevated mortality rate ranging from 40% to 87%^[4]^. Abscesses are usually supra-tentorial, single, multifocal and multiloculated. Even with insidious and nonspecific clinical presentation, CNS abscesses typically exhibit multiple concentric rims in T2-weighted MRI^[5]^.

Currently, the etiology is a gold standard for the diagnosis of *Nocardia* infection^[6]^. The involvement of the CNS in this patient indicated infection *via* hematogenous spread. However, positive findings of the organism in blood cultures are difficult. The aspiration of masses in the skin and soft tissues proved that the bacterium was *Nocardia*. Thus, not only blood but also the culture of secretions is suggested. However, most immunocompromised patients have some antibiotic therapy before microbial cultures for *Nocardia,* which reduces the positive rate of detecting *Nocardia*. Additionally, while most cultures of *Nocardia* species show positive results within a period of two to seven days, there are certain species that exhibit a slower growth. Consequently, the duration of the culture must be extended to two to three weeks^[2]^

In the case of immunosuppressed patients, a treatment regimen containing sulfonamide is recommended^[7]^, while other therapies, including minocycline, linezolid, ampicillin, amikacin followed by amoxicillin, and interferon gamma, have been reported^[8]^. The treatment duration lasts at least three to six months^[9]^. A combination of antimicrobials is commonly administered as an induction therapy in cases of severe nocardiosis because of the diverse antimicrobial susceptibilities that depend on the involved species^[10]^.

Therefore, we adjusted the treatments over time based on the results of antimicrobial susceptibility tests. The recommended first-line therapy for brain nocardiosis is TMP-SMX, which has the ability to cross the blood-brain barrier. Initial parenteral doses of 15 mg/kg of TMP and 75 mg/kg of SMX should be administered for a minimum period of three to six months.

Multi-agent intravenous therapy, including imipenem and cefotaxime or amikacin and TMP-SMX, has demonstrated successful outcomes in patients with a disseminated disease or a CNS involvement. In cases of severe nocardiosis, the addition of a third-line agent, such as linezolid, may provide further benefits. It is recommended that immunosuppressed patients with nocardiosis involving the CNS undergo at least 12 months of an antimicrobial therapy, followed by a regular follow-up^[11]^.

The patient in the present study was cured, although the mortality rate of patients with disseminated nocardiosis involving CNS infection is high. We are examining potential factors contributing to this observed success. Firstly, a prompt and accurate diagnosis of *Nocardia* infection was made within one week of the patient's admission. Secondly, a combination approach was adopted, involving the prolonged and standardized use of antibiotics that demonstrated sensitivity to the infection. Thirdly, in cases where the patient experienced severe conditions, such as CNS involvement, cardiac arrest, or convulsions, a prompt treatment was administered. Currently, there are more advanced diagnostic methods available for the detection of *Nocardia*, in comparison to bacterial cultures. These include next generation sequencing and matrix-assisted laser desorption/ionization time-of-flight mass spectrometry. These methods offer an increased sensitivity and timeliness in their ability to detect *Nocardia*.

In addition, the elevation of CRP from March 26 to April 15, 2017 could be attributed to the worsening of her infection with coma, sudden respiratory and cardiac arrest on April 15, 2017. Subsequently, a decrease in CRP levels was observed. The levels of SCr remained within normal parameters from March 25, 2017 to October 10, 2017. However, it exhibited a decrease on March 25, 2017, because of a severe infection that suppressed her appetite. From April 2016 to March 2017, the patient experienced a weight loss of approximately 25 kg, which may substantially decrease her muscle content and SCr level. Furthermore, we monitored lymphocyte subsets and counts throughout the treatment to observe the patient's immune function for the purpose of preventing infections.

## SUPPLEMENTARY DATA

Supplementary data to this article can be found online.
