# Supplementary material for: Successful rescue of disseminated Nocardia infection with multiple abscesses in a patient with membranous nephropathy after cardiopulmonary resuscitation: A three-year follow-up
Source: J Biomed Res. 2024 Jan 25;38(2):189–94. doi: 10.7555/JBR.37.20230107 (PMC11001588; doi:10.7555/JBR.37.20230107)
Supplement: Supplementary file 1 — Supplementary data to this article can be found online. [file jbr-38-2-189-S1.pdf]

Available online at www.jbr-pub.org.cn

Open Access at PubMed Central

Journal of Biomedical Research, 2024 0(000): 1–2

Supplementary Data

JBR

Successful rescue of disseminated *Nocardia* infection with multiple abscesses in a patient with membranous nephropathy after cardiopulmonary resuscitation: A three-year follow-up

Yili Xu[1,△], Hanyang Qian[1,2,△], Wen Qian[3], Li Dong[4], Weiying Liu[5], Yan Zhu[5], Yaning Mei[6], Yi Xu[3], Ling Wang[1], Yi Xia[7], Xu Qi[7], Huanping Mei[8], Xueqiang Xu[1], Huijuan Mao[1], Changying Xing[1], Ningning Wang[1,✉]

[1]*Department of Nephrology, the First Affiliated Hospital of Nanjing Medical University, Jiangsu Province Hospital, Nanjing, Jiangsu 210029, China;*
[2]*Department of Nephrology, Nanjing Tongren Hospital, Nanjing, Jiangsu 211102, China;*
[3]*Department of Image, the First Affiliated Hospital of Nanjing Medical University, Jiangsu Province Hospital, Nanjing, Jiangsu 210029, China;*
[4]*Department of Infectious Disease, the First Affiliated Hospital of Nanjing Medical University, Jiangsu Province Hospital, Nanjing, Jiangsu 210029, China;*
[5]*Department of Nephrology, the Second Hospital of Nanjing, Nanjing, Jiangsu 210029, China;*
[6]*Department of Microorganism, the First Affiliated Hospital of Nanjing Medical University, Jiangsu Province Hospital, Nanjing, Jiangsu 210029, China;*
[7]*Department of Hematology, the First Affiliated Hospital of Nanjing Medical University, Jiangsu Province Hospital, Nanjing, Jiangsu 210029, China;*
[8]*Department of Rheumatology and Immunology, the First Affiliated Hospital of Nanjing Medical University, Jiangsu Province Hospital, Nanjing, Jiangsu 210029, China.*

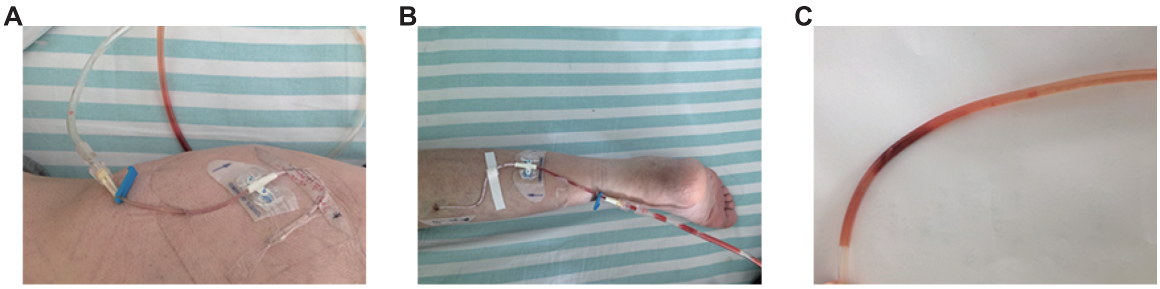

Supplementary Fig. 1 **Masses on the right buttock and in the popliteal fossa of the right leg on admission.** A and B: An egg-sized lump on the right buttock (A) and another in the popliteal fossa of the right leg (B) appeared and became enlarged after admission. C: Viscous pyogenic effusions were extracted from the lumps following intubation.

△These authors contributed equally to this work.

✉Corresponding author: Ningning Wang, Department of Nephrology, the First Affiliated Hospital of Nanjing Medical University, Jiangsu Province Hospital, 300 Guangzhou Road, Nanjing, Jiangsu 210029, China. E-mail: wangnn@njmu.edu.cn.

Received: 23 April 2023; Revised: 25 September 2023; Accepted: 07 October 2023; Published online: 25 January 2024

CLC number: R692, Document code: B

The authors reported no conflict of interests.

This is an open access article under the Creative Commons Attribution (CC BY 4.0) license, which permits others to distribute, remix, adapt and build upon this work, for commercial use, provided the original work is properly cited.

© 2024 by Journal of Biomedical Research.

https://doi.org/10.7555/JBR.37.20230107

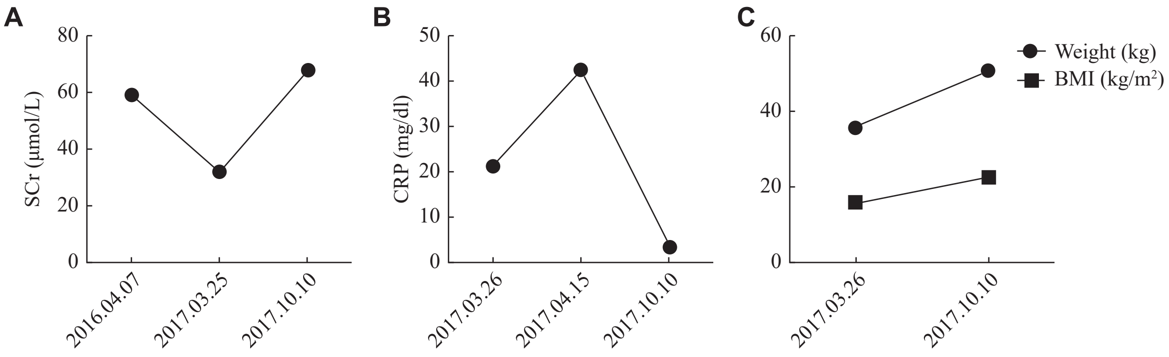

Supplementary Fig. 2 **Changes in biochemical indicators during the treatment and at a 6-month follow-up.** A: The levels of serum creatinine (SCr) were examined on April 7, 2016, March 25, 2017, and October 10, 2017. B: The levels of C-reactive protein (CRP) were examined on March 26, 2017, April 15, 2017, and October 10, 2017. C: The changes of weight and BMI between March 26, 2017 and October 10, 2017.

Supplementary Table 1 **The therapeutic schedule for the patient**

Date	2017.1.29–2.13	2.14–2.15	2.16–2.23	2.24	2.25–3.20	3.24–3.30
Immunorepressants	Withdraw					
Steroids	Methylprednisolone 16 mg/d					
Anti-bacterial	Moxifloxacin 0.4 g qd	Azithromycin 0.5 g qd		Linezolid 0.6 g bid	Levofloxacin 200 mg qd	
	Piperacillin tazobactam 3.39 g q8 h	Piperacillin tazobactam 4.5 g bid	Panipenem betamilon 500 mg q8 h		Amoxicillin sodium and sulbactam sodium 3 g bid	
Anti-fungal	Voriconazole 0.1 g q12 h					
Situation	External hospital					
Symptom	Cough, expectoration, persistent high fever	One lung lesion improved and the other did not	Persistent high fever		Normal temprature	Lumps appeared
Date	3.31–4.3	4.4–4.6	4.7–4.10	4.11–4.14	4.15–4.22	4.23–to date
Immunorepressants	Withdraw					
Steroids	Methylprednisolone 16 mg/d				Methylprednisolone 4 mg/d	
Anti-bacterial	TMP-SMX 0.96 g am. 0.48 g pm.	TMP-SMX 0.96 g am. 0.48 g pm.	TMP-SMX 0.96 g bid	TMP-SMX 0.96 g tid	TMP-SMX 0.96 g qid	TMP-SMX 0.96 g tid
		Azithromycin 0.5 g qd	Imipenem and cilastatin sodium 1 g ivd q8 h		Imipenem and cilastatin sodium 1 g ivd q8 h, linezolid 0.6 g bid, minocycline 50 mg bid	Imipenem and cilastatin sodium 1 g ivd q8 h, minocycline 50 mg bid
Anti-fungal	Voriconazole 0.1 g q12 h					
Situation	Our hospital					
Symptom	Puncture was conducted and revealed *Nocardia*				A sudden respiratory and cardiac arrest	Stable condition

Abbreviation: TMP-SMX, trimethoprim-sulfamethoxazole.
